# County-Level Social Distancing and Policy Impact in the United States: A Dynamical Systems Model

**DOI:** 10.2196/23902

**Published:** 2020-12-23

**Authors:** Kevin L McKee, Ian C Crandell, Alexandra L Hanlon

**Affiliations:** 1 Center for Biostatistics and Health Data Science Virginia Tech Roanoke, VA United States

**Keywords:** pandemic, SARS-CoV-2, infection control, COVID-19, social distancing, lockdown, nonpharmaceutical interventions, public health, intervention, model, infectious disease, policy

## Abstract

**Background:**

Social distancing and public policy have been crucial for minimizing the spread of SARS-CoV-2 in the United States. Publicly available, county-level time series data on mobility are derived from individual devices with global positioning systems, providing a variety of indices of social distancing behavior per day. Such indices allow a fine-grained approach to modeling public behavior during the pandemic. Previous studies of social distancing and policy have not accounted for the occurrence of pre-policy social distancing and other dynamics reflected in the long-term trajectories of public mobility data.

**Objective:**

We propose a differential equation state-space model of county-level social distancing that accounts for distancing behavior leading up to the first official policies, equilibrium dynamics reflected in the long-term trajectories of mobility, and the specific impacts of four kinds of policy. The model is fit to each US county individually, producing a nationwide data set of novel estimated mobility indices.

**Methods:**

A differential equation model was fit to three indicators of mobility for each of 3054 counties, with T=100 occasions per county of the following: distance traveled, visitations to key sites, and the log number of interpersonal encounters. The indicators were highly correlated and assumed to share common underlying latent trajectory, dynamics, and responses to policy. Maximum likelihood estimation with the Kalman-Bucy filter was used to estimate the model parameters. Bivariate distributional plots and descriptive statistics were used to examine the resulting county-level parameter estimates. The association of chronology with policy impact was also considered.

**Results:**

Mobility dynamics show moderate correlations with two census covariates: population density (Spearman *r* ranging from 0.11 to 0.31) and median household income (Spearman *r* ranging from –0.03 to 0.39). Stay-at-home order effects were negatively correlated with both (*r*=–0.37 and *r*=–0.38, respectively), while the effects of the ban on all gatherings were positively correlated with both (*r*=0.51, *r*=0.39). Chronological ordering of policies was a moderate to strong determinant of their effect per county (Spearman r ranging from –0.12 to –0.56), with earlier policies accounting for most of the change in mobility, and later policies having little or no additional effect.

**Conclusions:**

Chronological ordering, population density, and median household income were all associated with policy impact. The stay-at-home order and the ban on gatherings had the largest impacts on mobility on average. The model is implemented in a graphical online app for exploring county-level statistics and running counterfactual simulations. Future studies can incorporate the model-derived indices of social distancing and policy impacts as important social determinants of COVID-19 health outcomes.

## Introduction

As of September 8, 2020, the World Health Organization reports that the COVID-19 pandemic has resulted in about 900,000 deaths worldwide [1]. Through rapid government response and widespread changes in public behavior, countries including South Korea, Vietnam, and New Zealand have limited the spread of the virus and brought the number of new cases each day down to single digits. In the United States, 6.5 million cases have been detected, with more than 190,000 deaths; there were about 25,000 new cases and 286 new deaths on September 8 [2]. The demographic contributions to the United States’ mortality rate are not uniform. Those at greatest risk of mortality from the virus include older adults, those with existing medical conditions [3], and minorities [4,5].

Social distancing remains a basic and essential step toward limiting the spread of the virus [6], which has so far been found to transmit primarily in aerosols [7,8]. The United States has shown variation in regional and individual patterns of social distancing [9-11] in association with factors such as income [12] and political viewpoint [13-15]. In a survey study of 7355 people by Moore et al [16], 39.8% of participants reported noncompliance for reasons that included maintaining employment, mental health concerns, belief in sufficient precautions, childcare, distrust of media, and lack of direction. In many places, people began social distancing as soon as positive results came to light across the country and testing overall remained limited and inaccessible. Elsewhere, social distancing behavior has been practiced as a result of public policy, and in some cases, has been practiced very little or not at all [17].

Evaluation of policy impacts during the pandemic is complicated by a number of factors. Policies could be considered impactful either if subsequent patterns in public behavior correspond to the intentions of the policy, or alternatively by whether observable changes in public behavior occur at or after the time of the policy’s implementation. These represent two distinct choices of conceptual definition and statistical modeling approach. Currently, policy impacts during the COVID-19 pandemic have been evaluated with relatively rough linear models over a coarse statewide scale; these do not account for the dependency of observed changes and other, longer-term dynamics of public behavior [10,18,19]. Advanced time-series analysis modeling strategies are needed for the following reasons: (1) to account for social distancing behavior before the implementation of policy, based on information, public perceptions of risk, and early actions by institutions, (2) to incorporate information from all data points across time in a continuous manner rather than an arbitrarily selected number from before and after the policy date, and (3) to account for variable dynamic properties of mobility unique to each county in the response pattern.

New data on social distancing behavior per county has been derived by averaging over individual distancing metrics [20,21]. Such metrics are computed from the Global Positioning System (GPS) data obtained from individual mobile devices by cellular service providers. Social distancing metrics produce time series of public behavior across days and enable the study of their dynamics with respect to information, policy, and other rapidly changing factors related to the pandemic.

With high-resolution time-series data, dynamical systems models may be applied to estimate the dynamics, or time-invariant properties, of change in mobility and momentary changes due to particular events and policies. Of specific interest in this study are equilibrium dynamics, by which the mobility curve trends toward a certain point of normality over time. As economic and social pressures mount, we anticipate that demographics will vary in the rates at which they return to normal social behaviors. Momentary changes in policy can be jointly estimated with such dynamics to give fully integrated insights into their effects on the long-term trajectories of behavior. In this study, we operationalize policy impact by the degree to which social distancing practices are accelerated at the time of implementation conditional on the other social distancing dynamics in play.

We aim to do the following: (1) specify and statistically estimate the parameters of a dynamical system describing county-level mobility patterns, (2) use the model to estimate the effects of policy on long-term changes in behavior, and (3) examine the distribution of mobility dynamics and policy impact across counties. County-level analyses with the model are available through an online graphical R Shiny app, with the URL and further information in Multimedia Appendix 1.

## Methods

### Data

***Policy Data***

The main predictor variables of interest are the dates on which states put one of several mobility-restricting policies into effect. The policies considered here are stay-at-home (or shelter-in-place) orders (SAH), restrictions on all large gatherings (AG), closure of education facilities (EF), and closure of all businesses (AB). Unlike all other variables, policies are considered at the state level. Data on policy events are collected by the Kaiser Family Foundation [22].

#### Mobility Data

To model social distancing behavior, we used mobility data from Unacast [20]. Unacast uses displacement data from cell phone GPS averaged at the county level to give an index of mobility change. The measure for a given day is the percent change of mobility measured on that day from a baseline reference value calculated by Unacast from the period of February 9 to March 8, 2020. The baseline value was chosen by Unacast to represent average mobility levels from before any changes due to the pandemic. In total, 3 measures of mobility were used in this analysis. The first is the percent reduction from baseline in total distance traveled per device, averaged across all devices located in a county. The baseline for a given weekday is the mean distance traveled by the devices in the given county across four prepandemic weekdays of the same day. The second metric is the percent reduction in visitation to nonessential points of interest on a specific weekday compared to baseline as in the first metric. Points of interest include a variety of locations such as restaurants, department stores, hotels, and various other nonessential commercial locations. The third metric is the percent reduction in the rate of human encounters compared to the prepandemic baseline for a given county. Unlike the first two metrics, the baseline is the national average rate of human encounters taken over the 4 weeks prior to March 8, 2020, not the number of encounters on the same weekday. Any two devices are said to have an encounter when they come within 50 meters of one another within 60 minutes of one another. Each index was obtained for all days starting from February 24, 2020, and ending with May 31, 2020. No new, identified, human subjects data were collected for the present study, nor were mobility data available at an identifiable, individual level. Only county-level averages were available and used.

#### Covariates

Two county-level covariates, population density and median household income, were used to illustrate and validate the model by determining whether the estimated parameters reproduce previous findings and bear relation to relevant county-level attributes. Population density data were provided by the 2010 US census [23] and median household income data were provided by the 2016 US census. Population density was natural log–transformed for normality.

While our measures of population density are not the most recent and are expected to have changed since 2010, the locations of counties and urban centers along their distribution of density are unlikely to change enough to affect our results. We assume that most cities, suburbs, and rural areas have remained classifiable as such.

### Model

The model of social distancing behavior describes a smooth trend of mobility, M(t), characterized by changes in acceleration due to policy *Ψ_i_*, and information or other events *F_j_(t)*.


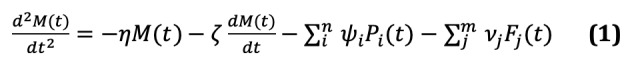


The parameters *η* and *ζ* together characterize the behavioral dynamics, including the rate of return to baseline social activity and social inertia. Specifically, the further *M(t)* strays from the baseline value of 0, the larger its acceleration will be in the opposite direction. The total acceleration is also proportional to the instantaneous velocity, such that the system moves in a more or less “viscous” manner. This model is known in engineering and mathematical literature as the damped linear oscillator. The parameter *η*, when greater than 0, is the squared frequency of oscillations around the equilibrium point, while *ζ* determines the damping or decay in amplitude of those oscillations. The half-period in days given by *η* with adjustment for *ζ* is 
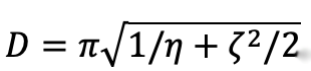
. For critically damped or overdamped systems, this expectation will not correspond to the zero-crossing of *M(t)*, as the zero value in those cases is the asymptote of the trajectory. The parameter conversion is nonetheless useful for comparing overall rates of return.

When 
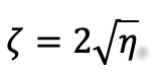
, the system is critically damped, meaning oscillatory behavior vanishes and the expected return to equilibrium is monotonic. At 
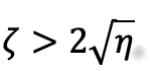
, the overall rate of monotonic decay toward equilibrium is further reduced. A dimensionless index of social inertia will be given in this case as 
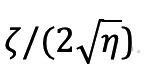
, which is conventionally known as the damping ratio. In physics, *η* is often used to represent a spring constant, gravitational force, or the strength of any kind of attractor, while *ζ* is used to represent forces of friction, viscosity, or other factors in the dissipation of energy. We chose this model for its analogous interpretation when applied to the mobility data. The frequency parameter *η* in this case represents the total forces driving public behavior toward its normal baseline, including the need to work and maintain employment and desire for normal social interactions. We interpret it as the average eagerness to return to normalcy, and from that we can compute a predicted number of days until public mobility has returned to its normal rates. Regarding friction or viscosity, the best analogy may be a tendency toward social inertia by which people are resistant to both the initial reductions in mobility as well as later reversions to normalcy. We continue to refer to this as damping in the current study but note this possible interpretation. As with a physical system, the damping parameter determines how gradually people returned to baseline, if they did at all. For small values of damping, we expect to see higher than normal levels of mobility following the period of social distancing.

As we are fitting the above model to multiple noisy indices of mobility, a measurement model is necessary. This part of the model brings the indices onto a common scale and allows them to be described altogether by the dynamics of *M(t)*. The indices, ***y****(t)*, are proportional to *M(t)* plus additional, normally distributed noise or short-term fluctuations, ***v****(t)*. The residual covariances of the indices, Σ, is a diagonal matrix (ie, the residuals are assumed to be uncorrelated).


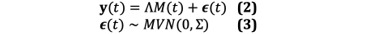


**Figure 1:**
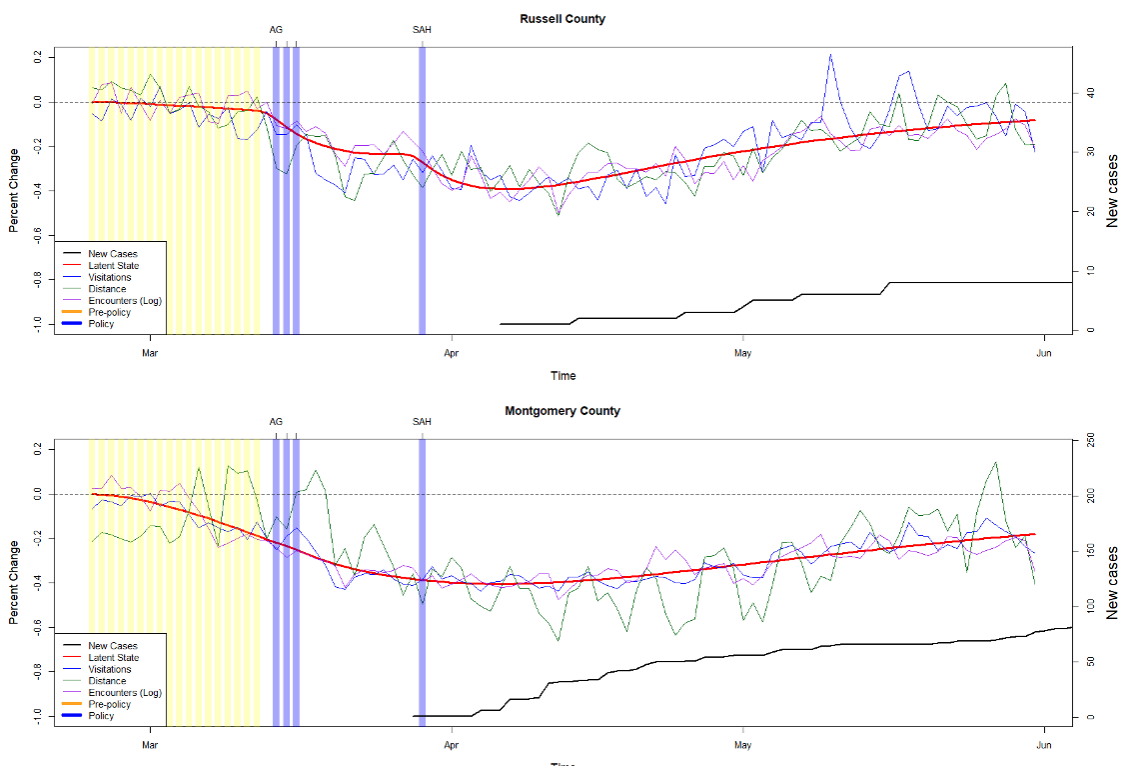
Two examples of social distancing behavior by county with estimated dynamics and policy effects. Top: One county shows no pre-policy distancing followed by inflections at the dates of major policies. Bottom: Distancing was driven almost entirely by pre-policy behavior; policy had little to no effect. The Virginia policies shown in order were restrictions on all large gatherings (AG), closure of all businesses (AB), closure of all educational facilities (EF), and stay-at-home orders (SAH).

The column vector of weights Λ includes one weight fixed at a value of 1.0 to anchor the scale of *M(t), Λ* = {1, *λ_2_*, *λ_k_*}. The parameter for visitations was chosen to be fixed such that *M(t)* took on the scale of percentage change. Two examples of the complete model fit to data are given by Figure 1, with the red line showing the estimated trajectory of *M(t)* overlaid on scaled distance traveled (green), visitations (blue), and log encounters (purple). The pre-policy period is highlighted in yellow, and individual policy dates are shown as the blue vertical lines.

A formal model of the distributions of parameters *η* and *ζ* and their functional mappings from demographics is left to future work.

### Outputs

Table 1 gives a summary of the model outputs with descriptions of each, including transformations. Indices labeled “damping ratio” and “approximate days until normal” are useful because the transformations eliminate systematic correlations between *η* and *ζ*, and thus represent unique features of the resulting trajectory of *M(t).* Policy effects *Ψ_i_* are computed for the 4 policies abbreviated AB, AG, SAH, and EF. If two or more policies were enacted on the same day, the model was not identified and the effects of each had to be grouped into a single “unknown” policy parameter *F(t)* that represents pre-policy distancing, which is formalized as a continual force on *M(t)* distributed over all the days leading up to the first state policy. Rhode Island did not issue any policies. All other states enacted at least two policies, with all closing educational facilities, all except North Dakota restricting all gatherings, and all except South Dakota closing all businesses.

**Table 1:** Parameters of the model.

Parameter	Description
*η*	Frequency, rate of return to normality
*ζ*	Damping, social inertia
*R* = 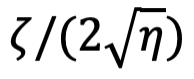	Damping ratio (0=undamped, 1=critically damped)
*D* = 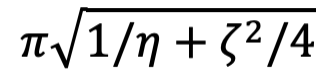	Period, expected days until normal
*Ψ_i_*	Policy effect *i*
*v_i_*	Pre-policy distancing, other effects *j*

### Estimation and Software

The model was fit to each county time series of mobility indices using Maximum Likelihood Estimation (MLE) in an R statistical coding environment [24] (R Foundation for Statistical Computing) using the OpenMx package [25]. Statistical models were specified as continuous-time state-space models with Kalman-Bucy filter implementation [26,27]. Optimization of model parameters to obtain statistical estimates was performed with gradient descent, followed by computation of standard error from numeric derivatives. Parameters were constrained such that *Ψ_i_, v_j_* ≥ 0 and *η* >0.

## Results

### Quality Control

Estimates were obtained for a total of 3050 counties and cities in the United States. In total, 4 counties were removed for extreme parameter estimates with *D*≥500 or *R*≥100: Northwest Arctic, Alaska; Clark, Arkansas; Eagle, Colorado; and Fillmore, Nebraska. Mobility data for these counties resembled random noise, with rapid fluctuations around a fixed mean value. As a result, dynamic parameters were likely unidentified, thus trending toward extremely high values (ie, frequency of zero, equivalent to infinite number of days until normal or no oscillation). States without certain policies were marked as missing data for the parameter estimates representing the respective policies’ effects (Table 2). All states except North Dakota issued AG, and all except South Dakota issued AB. Only 12 states did not issue a SAH order.

**Table 2:** Percentages of policy effects across counties with estimated near-zero effect and percentage implemented (N=3139).

Policy	Counties where implemented, n (%)	Near-zero effect, n (%)	Number of counties	Number of states
Stay-at-home order	1939 (61.77)	566 (18.03)	1938	38
Ban on all gatherings	1440 (45.87)	411 (13.09)	1436	49
Closing all businesses	1506 (47.98)	467 (14.88)	1502	49
Closing of educational facilities	2381 (75.85)	544 (17.33)	2377	50
Unknown (overlap)	1759 (56.04)	620 (19.75)	1758	30

### Descriptive Statistics

Descriptive statistics were obtained for each of the dynamics and policy effects estimated by the model and are shown in Table 3. The most commonly implemented policy was the closure of educational facilities, though for many counties its effect was grouped under “unknown” because it occurred on the same day as one or more other policies, making the number of estimated effects fewer than the total number of counties. From the frequency parameters, the average expected days until normal was estimated to be about 62, with 95% of estimates falling between 28 and 96 days. The damping ratio varied highly, with an average close to the critical damping value of 1, with outliers at 0 (no damping) and 24. Counties with a high damping ratio showed little or no acceleration toward normal but rather a very gradual and nearly linear trajectory.

**Table 3:** Descriptive statistics for all social distancing and policy parameters.

Parameter	μ	σ	Median	Minimum	Maximum
D (period, return rate)	62.615	17.101	62.427	12.406	141.981
R (damping ratio, social inertia)	0.949	0.793	0.816	0.000	24.184
*η* (raw)	0.004	0.020	0.003	0.000	1.093
*ζ* (raw)	0.128	0.932	0.081	0.000	50.570
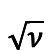 (pre-policy)	0.116	0.070	0.120	0.000	0.893
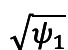 (stay-at-home)	0.083	0.089	0.077	0.000	0.958
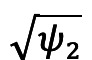 (ban on all gatherings)	0.085	0.081	0.089	0.000	0.323
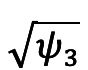 (closure of all businesses)	0.059	0.074	0.001	0.000	0.847
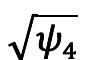 (closure of educational facilities)	0.086	0.092	0.083	0.000	1.517
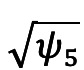 (unknown)	0.064	0.064	0.063	0.000	0.707

**Figure 2:**
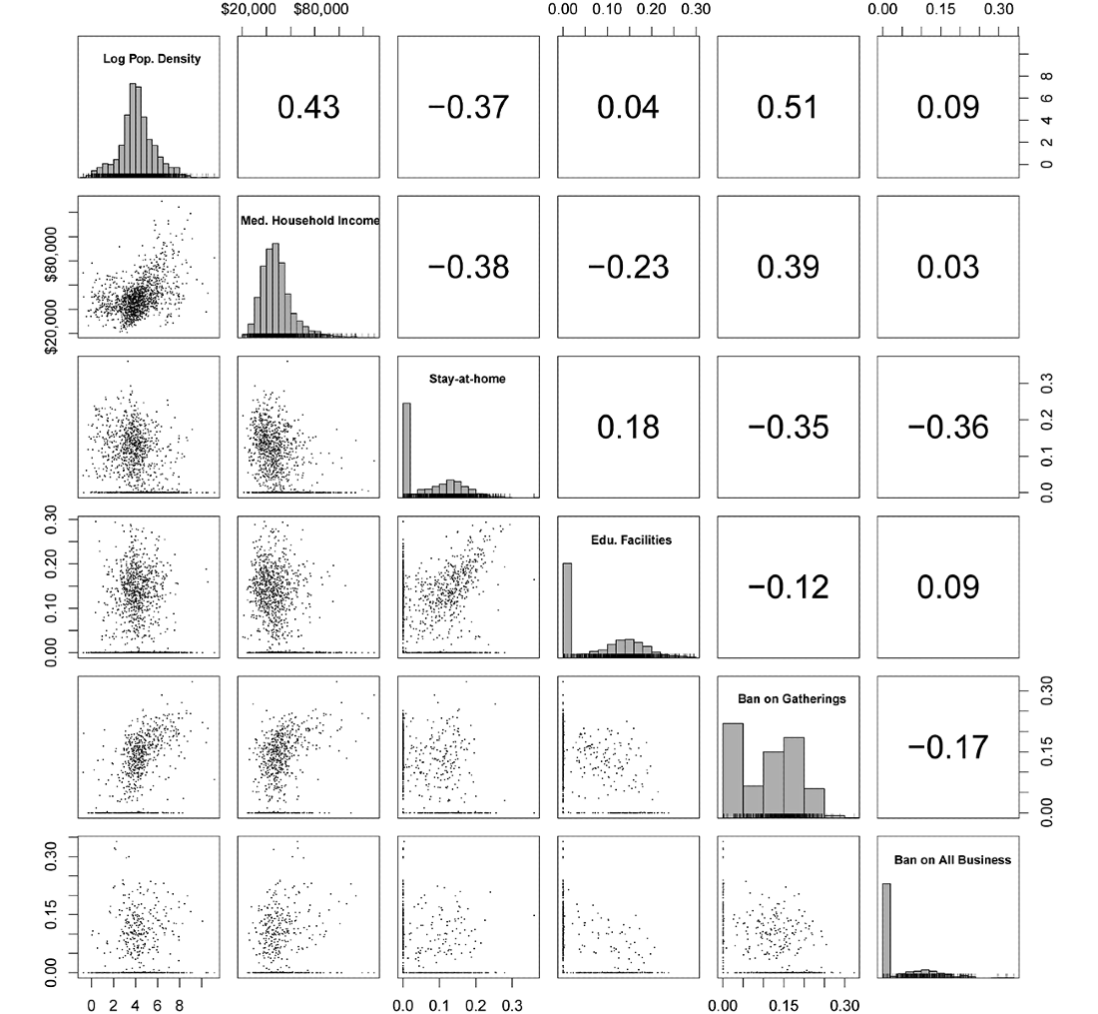
Scatter plots of county-level policy effects with two demographic covariates. Spearman correlations are given in the upper-right triangle and marginal histograms are shown in the diagonal cells.

**Figure 3:**
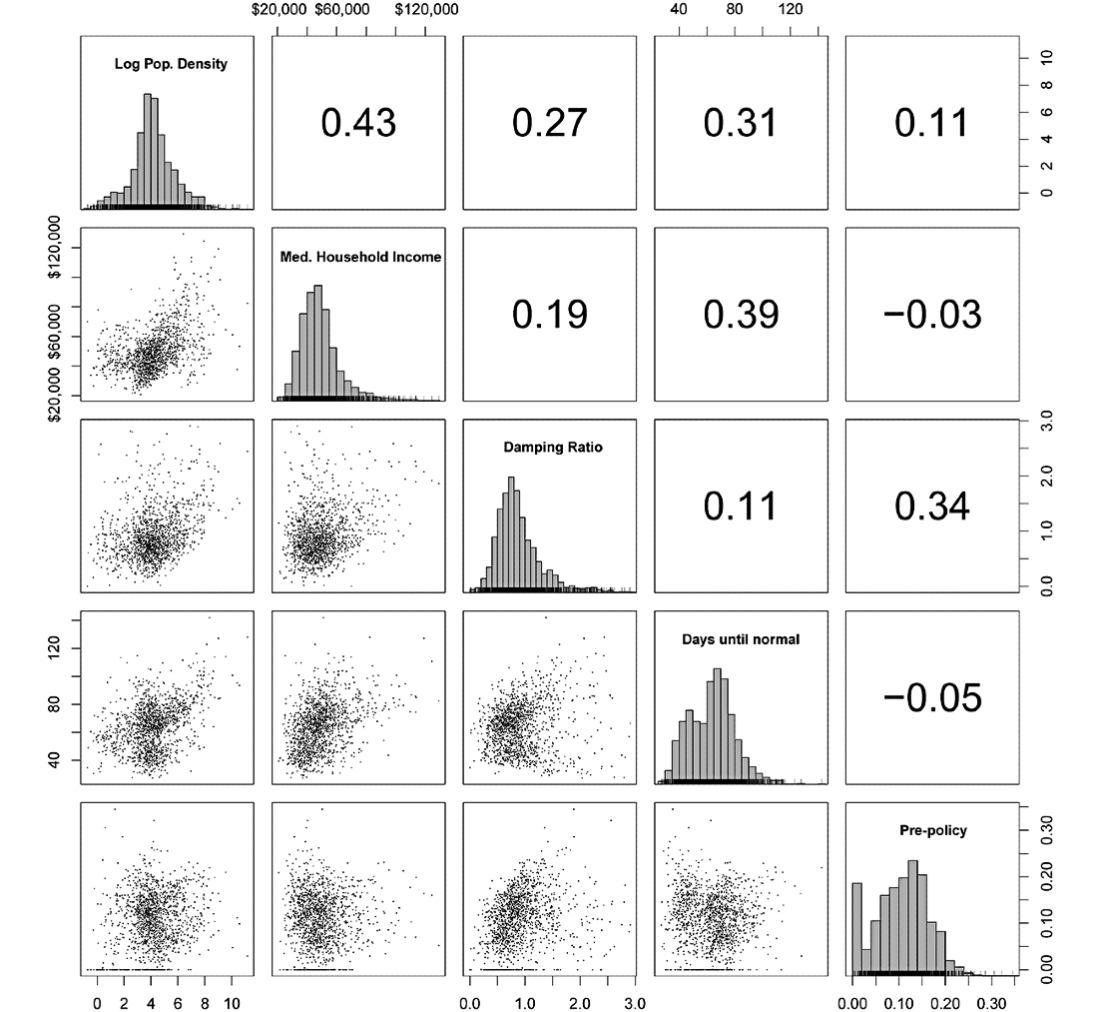
Scatter plots of county-level mobility dynamics with two demographic covariates. Spearman correlations are given in the upper-right triangle and marginal histograms are shown in the diagonal cells.

Figures 2 and 3 show the matrices of Spearman correlations between estimated policy effects, mobility dynamics, and two demographic covariates: the log population density and median household income. Policy (*Ψ*) and pre-policy (*ν*) social distancing effects were reverse coded to positive values and the square roots were taken to improve normality. The transformed frequency and damping (as expected days until normal and damping ratio, respectively) were used to eliminate parameter dependence.

The policy effects that correlated most with population density and median household income were SAH and AG (Figure 2). Both policies had moderate to large associations but with opposite signs: with Spearman *r*(SAH, log population density)=–0.37, 95% CI –0.42 to –0.33, *P*<.001; *r*(SAH, median household income)=–0.38, 95% CI –0.42 to –0.33, *P*<.001; *r*(AG, log population density)=0.51, 95% CI 0.46 to 0.56, *P*<.001; *r*(AG, median household income)=0.39, 95% CI 0.32 to 0.44, *P*<.001. More densely populated areas with lower median income complied more with AG and less with SAH. AB and EF showed only small associations. Few counties had nonzero effects for more than two policies.

Both the damping ratio and expected days until normal showed weak to moderate positive associations with population density and median household income (Figure 3). Pre-policy distancing was uncorrelated with household income (Spearman *r*=–0.03, 95% CI –0.08 to 0.02, *P*=.58) and had only a weak positive correlation with log population density (*r*=0 .11, 95% CI 0.06-0.16, *P*=.03). Household income had the strongest relationship with the expected number of days until normal (*r*=0.39, 95% CI 0.35-0.44, *P*<.001). Log population density was also associated with days until normal (*r*=0.31, 95% CI 0.27-0.36, *P*<.001). The correlations suggest that cities experienced the most gradual reversion to normal social behavior, with some maintaining nearly constant levels of social distancing up to the present day. The expected number of days until normal shows a bimodal Gaussian distribution. The subdistribution with the higher mean has an upper tail that was more correlated with population density than the rest of the distribution, possibly representing the comparatively small number of major cities in the sample. The same nonlinear relationship is apparent to a lesser extent in the joint distribution of expected days until normal and median household income, suggesting that social distancing behavior may be stratified by other relevant socioeconomic factors.

### Effects by Chronology

The chronological order of the policies (see Figure 1) may be a large determinant of their effects as estimated by the model. To test this, Spearman correlations were calculated between the effect size and chronological rank order per county of each policy. All correlations of effect size with rank order were negative and moderate to strong except for the closing of businesses. From Table 3 and Figure 2, we can see that the small correlation of business closings is likely due to its overall small effect in most counties. SAH was implemented later than all other policies on average, while AG tended to be the first and most effective policy. For each policy, there were many zero-valued estimates, partly as a result of the precedence of other policies, as shown in Table 2. Zero-valued estimates were distinct from missing estimates, which were due to lack of implementation in certain states.

The chronology of state policies helps to explain the correlations between their effect sizes (Figure 2). The results given in Table 4 suggest that per county, the maximum total change in mobility was distributed unequally between the four policies. Where an earlier policy accounts for most of the changes, later policies were less consequential. This was true for pre-policy distancing as well. Pre-policy distancing was weakly negatively associated with the earlier AB and AG, but positively associated with the later SAH and EF.

**Table 4:** Spearman correlations of policy effect sizes with chronological rank order of implementation.

Policy	*r* (95% CI)	*μ* Order
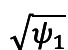 (stay-at-home order)	–0.33 (–0.37 to –0.29)	3.30
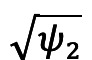 (restriction of all gatherings)	–0.56 (–0.60 to –0.53)	1.51
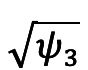 (closure of all businesses)	–0.12 (–0.17 to –0.07)	2.08
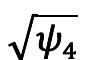 (closure of educational facilities)	–0.42 (–0.45 to –0.39)	1.77
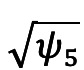 (unknown)	–0.37 (–0.41 to –0.33)	1.70

## Discussion

### Principal Findings

In this study, we used three indices of social distancing behavior and evaluated four social distancing policies across the United States. Many of these indices have moderate to strong correlations with two demographic variables, population density and median household income, which are themselves strongly correlated. These results, though descriptive, suggest that there are notable differences between cities and rural areas in the average degree to which people in each complied with the social distancing policies and practiced distancing before the first policy was issued. We also demonstrated differences in the long-term social distancing behavior using indices of the expected time taken to return to normal, computed from the model frequency parameter, and the general responsiveness to policies and information represented by the damping ratio parameter. Our results support previous findings that the SAH (or shelter-in-place) order and EF (or educational facility closings) were the most influential, but contradict the finding that AB, the ban on gatherings, had a smaller effect [10,28,29].

Population density was more strongly associated with AG than SAH. One possible explanation is that large-scale public gatherings are generally more common in urban centers than rural places, and there are more available venues, whereas in rural communities, people may tend to gather in smaller groups at home. Conversely, household income had the same magnitude of association with both AG and SAH. The association with income aligns with other recent findings that wealthier individuals often have jobs that allow them to work from home and control their immediate environment in other ways [12,30-32].

The SAH order and AG had oppositely signed associations with both population density and household income. They were also negatively correlated with each other. The overall finding that unites these statistics is that SAH and AG seemed to apply to different populations, with SAH delineating the time of the largest rural changes in mobility, and the AG applying mainly in urban centers.

Two social distancing dynamics, damping ratio and days until normal, were also associated with population density and income. The relationship is easily observed by browsing the graphical Shiny app interface for our analysis [33], with rural counties showing comparatively smaller changes in mobility and sharper curves in the long-term trajectories back toward baseline. There may be many related reasons for the differences by both population density and household income, including less job security and financial stability over longer periods of distancing [12,32], political differences that correlate with the given measures of income and density [13,14,34,35], and differing access, trust, or comprehension of scientific and medical information about the pandemic [36,37].

It is also apparent from our analysis that the chronological order of policies was strongly related to their impact, suggesting that policy order is more important than policy content for slowing mobility. Among similar policies like AG and SAH, it may not have mattered which policy was chosen but only which came first. The first response from the state acted as an official determination of individual risk levels beyond information conveyed by social media, doctors, or scientists. Possibly, many people made up their minds at that point about the necessary precautions to take, and further policies were redundant toward their personal decisions. Additionally, subsequent policies may have lost influence if it became apparent that the initial policy was not sufficiently enforced. It is clear in any case that states will benefit from taking decisive and early action in the future as soon as the risks become clear.

The prominence of pre-policy effects implies that early informational campaigns were in many places more important than particular policies, but our analysis did not find this effect to be strongly stratified by population density or at all by income. Pre-policy and other nonpolicy effects may have also resulted from universities and employers taking early action regardless of state orders. Armstrong et al [38] devised a measure of policy “aggressiveness” and found an overall association with reductions in mobility and lower rates of COVID-19 deaths but conclude that lower rates of distancing in Canada are not well explained by policy alone. They also note some of the unmodeled differences in the long-term trajectories of mobility among cities that were captured by our own model, such as whether mobility remained low, regardless of initial response. The three aforementioned studies of policy impact likewise found a significant role of voluntary or otherwise non–policy-related changes in mobility [10,28,29].

### Limitations

As information about and management of the pandemic have been highly politicized, and oversimplified analyses stand to negatively impact vulnerable communities, we refrained from undertaking a more complete observational analysis of demographic factors contributing to our observed trends in mobility. Rather, future work to determine the causes of different social distancing behaviors will have to carefully consider the complex and tightly intercorrelated nature of socioeconomic, racial, geographic, educational, and political factors. We considered a sufficiently responsible analysis of other demographic factors beyond the scope of this study.

For similar reasons, we chose to limit our analysis to the effects of policy on social mobility rather than to extend it to case counts and deaths. While it is feasible that policy-induced reductions in mobility would have an effect on case counts and deaths, it is not possible to observe the counterfactual and measure what cases or deaths would have been without the policy. Further, taking account of the effects of policy on cases and deaths would necessitate the use of a comprehensive model of disease spread, which we considered to be beyond the scope of the present work. Rather, our aims were primarily methodological and focused on one aspect of the dynamic interplay between policy, mobility, and spread. From this work, we can offer both the mobility time series model and the indices derived from it as a supplement or basis for future work and collaboration.

Many counties had mobility damping ratios less than one. This means that they did not monotonically return to baseline but overshot and actually had above-average levels of mobility later in time. There are at least two possible explanations for this observation. On one hand, many counties may have legitimately reacted to the previous social distancing by “making up for lost time” and increasing their social activity or attending to business at more urgent rates.

Alternatively, the phenomenon may arise because indices of relative mobility were computed in reference to the February average, and seasonality over the rest of the year was not considered. This second explanation seems more likely as outdoor activity and travel increase throughout the spring and summer. The latter scenario may account for some degree of the return behavior in all counties. Variable magnitudes of average seasonality by county are therefore an important confound to the behavioral dynamics estimated in this study. Other factors, including mask usage and systematic adaptations of normal business operations to pandemic circumstances, were not included in the model but likely account for some part of the public’s willingness to re-engage in social behavior later in time. It is also possible that the effects of chronology found in this study (ie, weakening of policy effects implemented later in their succession) result from the upward seasonal trends in mobility at that time. Arguably, the 2-week interval over which all policies were implemented in each state is too short to reflect seasonal changes observed over several months.

In this analysis, typical seasonal fluctuations were necessarily subsumed under the effect size of the frequency parameter, used to calculate the expected days to return to baseline. If counties experience similar degrees of seasonal fluctuation on average, then it is possible that seasonality induces only a mean affect across the frequency parameters, meaning relative comparisons based on the variation around that mean are still informative. However, this is not a strong assumption and future studies implementing this model should seek data sets that include the full year of 2019 to provide seasonal adjustments for mobility across 2020. Subtracting out monthly averages will ensure more accurate expectations of social and business equilibria. A free, open-source alternative source for such information may be, for instance, SafeGraph [39].

The indices *η* and *ζ* represent average tendencies of public behavior given the assumptions of the model. They do not necessarily represent anything intrinsic or invariant to each subpopulation. The model may therefore be improved by allowing for nonstationarity in these dynamics as new information, changes in policy, and the aforementioned factors of mask usage and business operations gradually change public behavior.

We also recognize that the model assumptions may not perfectly represent the “true” data-generating process, as they were chosen for generality and simplicity in describing the common patterns of change across all counties. There are plausible scenarios in which the assumptions of the model may not be valid. Policy changes were detected relative to the ongoing trajectory of social distancing, which was assumed to be continuous and differentiable, and to follow a linear second-order differential equation. However, policy impact could be defined differently and evaluated according to alternative models that do not make these assumptions. For instance, if the model expectation absent any policy effect is a sharp turn in mobility toward its baseline, then a smooth continuation of the trend away from baseline (ie, absence of any inflection point) would be modeled as a nonzero effect. It was therefore our own judgment that the presence of an inflection point along a smooth path was the most obvious indication of a policy effect.

### Future Directions

As the pandemic continues, the model may be periodically updated with new events, policies, or other covariates to account for continued changes in mobility. Positive tests for COVID-19 and counts of mortalities were not included in this model, but they are likely a factor in motivating increased social distancing behavior. An improvement to the model will therefore incorporate the dynamics of the disease itself to represent the real feedback relations between its spread and distancing behavior.

Demographic variables such as income, population density, political vote share, race, and so forth may explain variation in the rates of distancing and compliance with policy. Policies were unlikely to apply equally to different counties and subpopulations. People living in densely populated areas and with lower incomes may have been unable to avoid sharing spaces or were more likely to be deemed essential workers and thus prohibited from social distancing by their employers. Many of the previously mentioned covariates, including political vote share and race, are both highly controversial and complex. Simple correlations or linear regressions will not be sufficient to draw conclusions about their roles in the pandemic. The authors thus plan to incorporate the current derived data set into structural equation models and other appropriate strategies in subsequent work.

Our modeling approach provides a general method for evaluating the effects of one-time occurrences, whether policies or events, and may be useful for deciding the type and timing of future policies. Furthermore, counterfactual simulations may be run by using estimated model parameters as starting values, then adjusting the parameters according to counterfactual scenarios, such as testing the consequences of a later implementation date for a particular policy. As a tool for policy evaluation, counterfactual simulations will be particularly useful when future iterations of the model are specified to relate social distancing behavior to mortality and positive test results. Such simulations would allow empirical estimation of the number of deaths or infections prevented or preventable by particular events and policy decisions.

The general method used here may be applied at different scales insofar as GPS-based indices of mobility can be obtained. For example, the model may be applied to evaluate university-level policies intended to limit spread among students at the start of a new semester, determine the effects of possible spreading events, and further examine the co-evolution of public behavior with the spread and control of the pandemic.

### Conclusions

We have proposed a novel strategy for modeling changes in mobility during the COVID-19 pandemic, with a special focus on evaluating the impact of policies. Our approach relies on state-space modeling with differential equations to estimate the equilibrium behavior of public movement and impacts of particular events and policies. A preliminary observational result reveals that the chronology of the policies is a major determinant of their relative impacts. Substantial distancing was undertaken in many counties before any policy was implemented, pointing to the influence of other nongovernmental authorities and public information. Population density and median household income were associated with both policy impacts and long-term trends in social distancing, showing separate, stratified impacts. With this summary of the model and brief analysis, we provide in Multimedia Appendix 1 an online R Shiny application that allows examination of county-level data with model expectations and counterfactual simulations.

## Appendix

Multimedia Appendix 1 Supplementary figure.

## Abbreviations

AB: closure of all businesses
AG: restriction of all large gatherings
EF: closure of educational facilities
GPS: Global Positioning System
SAH: stay-at-home order
