# Supplementary material for: County-Level Social Distancing and Policy Impact in the United States: A Dynamical Systems Model
Source: JMIR Public Health Surveill. 2020 Dec 23;6(4):e23902. doi: 10.2196/23902 (PMC7759510; doi:10.2196/23902)
Supplement: Multimedia Appendix 1 [file publichealth_v6i4e23902_app1.docx]

## Multimedia Appendix 1

An online graphical interface for the mobility model is available online [33]. The app allows browsing of county-level mobility data, model expectations, policy times, and positive test results. Figure 4 is a screenshot of the app, showing map-based navigation, time-series plots, and the dates and impacts of each policy. The solid green, blue, and purple lines respectively display daily average distances traveled, number of visitations to key points of interest, and log number of encounters. These indices are re-scaled for display with the latent mobility state, in red, to show the similarity of their trajectories on a common scale. The vertical blue lines represent dates in which policies were issued, with the abbreviation for each policy shown above. Daily positive COVID-19 test results are shown in black, with the right-hand Y-axis.

On the left side, check-boxes allow both data and simulations to be displayed or hidden. The simulation function allows series to be generated at the estimated values of each county to examine counterfactual scenarios. Conditional on the settings of the simulated infection rate, the simulation also plots a green, horizontal, dotted line representing the threshold under which social distancing levels must pass to flatten the infection curve. The parameters of the simulation may be adjusted using the numeric input boxes on the left, either by directly entering numbers or in increments with the small arrow buttons. Currently, the parameters of the infection curve are not estimated but may be manually adjusted.


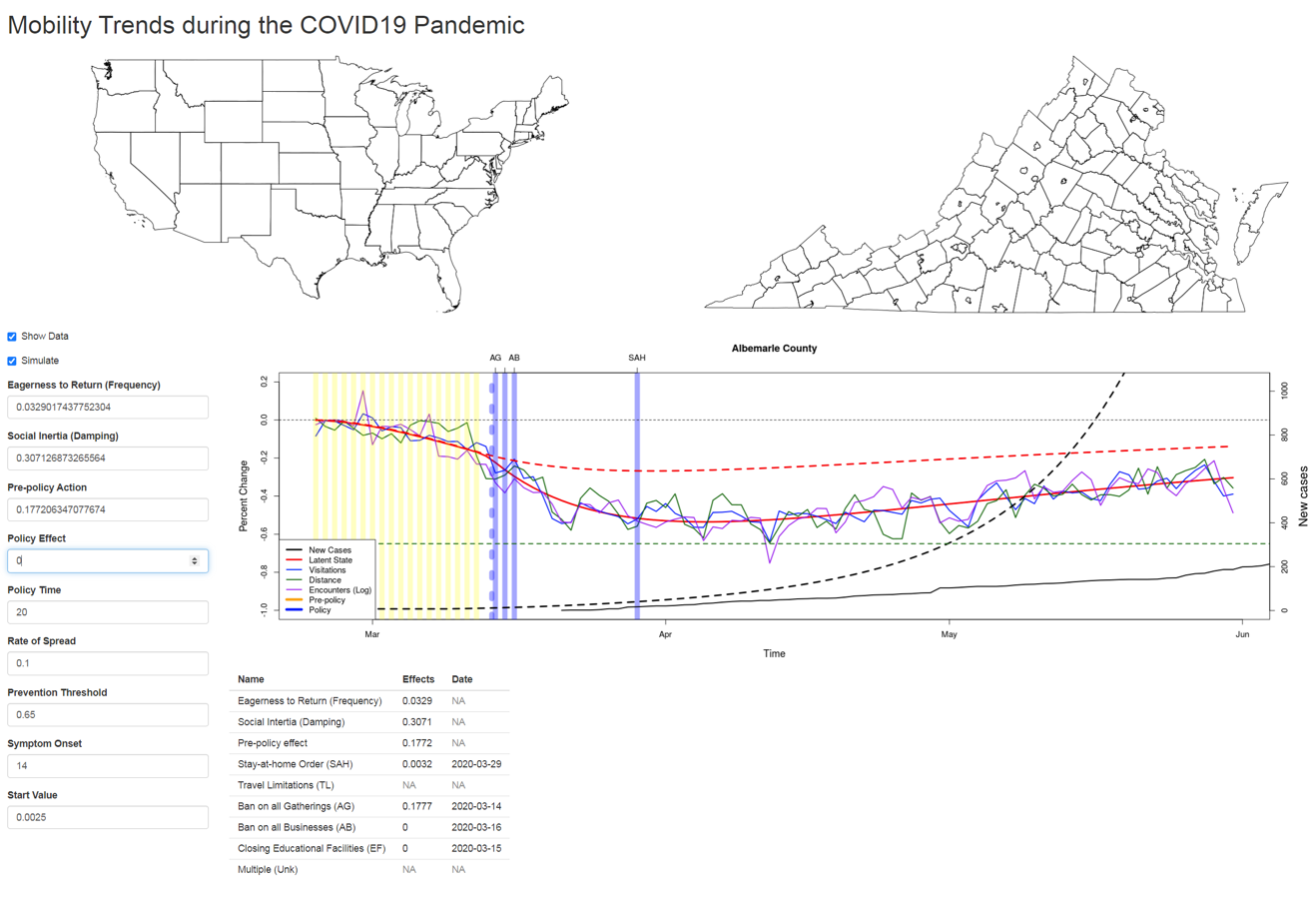


Figure 4: Screenshot of graphical online mobility app. County selection is done by clicking on states and counties on the maps. Simulation controls are on the left and model outputs are below the graph.
